# Case Report: Pott's Edematous Tumor: Complicated Frontal Sinusitis - An Unremembered Diagnosis

**DOI:** 10.3389/fsurg.2022.889463

**Published:** 2022-06-27

**Authors:** Ana Cristina Veiga Silva, Camila Mendonça Lins, Renan Furtado de Almeida Mendes, Marcelo Henrique Simões Silva, Joaquim Fechine de Alencar Neto, Caio César Maia Lopes, Gabriela Lisboa de Souza Ferraz, Diego Felipe Rodrigues de Sousa, Luiz Severo Bem Junior, Marcelo Moraes Valença, Hildo Rocha Cirne de Azevedo-Filho

**Affiliations:** ^1^ Department of Neurosurgery, Hospital da Restauração, Recife, Brazil; ^2^ Neuroscience Post-Graduate Program, Federal University of Pernambuco, Recife-PE, Brazil; ^3^ Faculty of Medical Sciences, University of Pernambuco, Recife, Brazil; ^4^ Center for Medical Sciences, Federal University of Paraíba, João Pessoa, Brazil; ^5^ Department of Pathology of the Medicine Faculty, University of Paulo, São Paulo, Brazil; ^6^ Faculty of Medical Sciences, Unifacisa university center, Campina Grande, Brazil; ^7^ Department of Radiology, Hospital da Restauração, Recife, Brazil

**Keywords:** Pott’s Puffy tumor, Pott’s edematous tumor, osteomyelitis, sinusistis, forehead swelling

## Abstract

Pott’s Puffy tumor, also called Pott’s edematous tumor (PET), is a subperiosteal abscess of the frontal bone, associated with osteomyelitis of the frontal bone. In this paper, we report the case of a 16-year-old patient who presented with headache associated with progressive forehead swelling and fever. Clinical and imaging exams pointed to the hypothesis of PET associated with brain abscess. Patient was submitted to surgical excision of the abscess and treatment of osteomyelitis, with intraoperative findings corroborating the condition. There was a good clinical-radiological recovery associated with prolonged antibiotic therapy and satisfactory follow-up after hospital. PET, which often results from an underdiagnosed or partially treated frontal sinusitis, is a condition that must be promptly recognized and directed to an adequate therapeutic approach due to the risk of serious complications that it entails.

## Introduction

PET is usually an extracranial complication of frontal sinusitis and is characterized as subperiosteal abscess of the anterior wall of the frontal sinus, associated with osteomyelitis of the frontal bone. It was first described by Sir Percival Pott, as a consequence of head trauma (1), however, it is known that currently the main causes are rhinosinusitis, acute or chronic, underdiagnosed or inadequately treated (2–4). PET may still be associated with other conditions such as drug use, infections and frontal surgeries (2).

Due to the modern age of antibiotics, it is a rare entity and occurs more frequently in adolescents, probably due to vascularization and abundant flow in the diploic veins in this age group, facilitating the spread of infection (3). Its most common clinical manifestation is localized frontal swelling accompanied by fever and headache (2).

Early diagnosis plays a decisive role in patients with PET, as it allows the execution of precise treatment before the onset of complications, such as epidural, cerebral abscesses, subdural empyema, meningitis, and cerebral venous thrombosis. The clinical history added to the radiological findings of computed tomography (CT) directs the diagnosis. In cases of suspected intracranial injury, magnetic resonance (MRI) is indicated (4).

We describe the case of an adolescent patient, complaining of headache and presenting with fever, vomiting and forehead swelling. The patient, without known comorbidities, developed an intracranial complication of an infrequent sinus infection. The findings reported were related to relevant data from the literature, emphasizing the management and the need for early diagnosis.

## Methods

This article is a case report with a literature review. Patient data and images were collected by the researchers who participated in the patient’s care. The consent form was signed, and the patient and guardians are aware of the possibility of writing and publishing the data and images present in the manuscript. The literature was reviewed by one of the researchers based on the search for articles in the PubMed database. For the research, the following keywords were inserted: “Pott puffy” and “tumor”, “Pott’s edematous tumor”. The “Boolean Operator” “AND” was used to maximize the amount of articles published. Inclusion criteria reached articles published in the last five years, case reports and patients aged up to 18 years. Thus, 49 articles were found based on the ones, of which 22 met the defined analysis criteria.

## Case Description

Female patient, 16-year-old, single, brown, student, born in rural area of Pernambuco/Brazil, referred to the neurology and neurosurgery service of the Hospital da Restauração with a history of frontal, pulsing and progressive headache, worsening over weeks, associated with emetic episodes and fever. The condition evolved with progressive growth of a painful, adhered and fibroelastic consistency mass in the central region of the forehead of the skull, eight days before admission. Report of fever stopped two days after admission, with no signs of meningism, rhinorrhea or any focal neurological deficit. No traumatic event, past or recurrence of sinusopathy or other comorbidity that was related. Patient also referred no use of antibiotics. Laboratory tests also did not present alterations of hematimetric indices or inflammatory tests.

**Figure 1:**
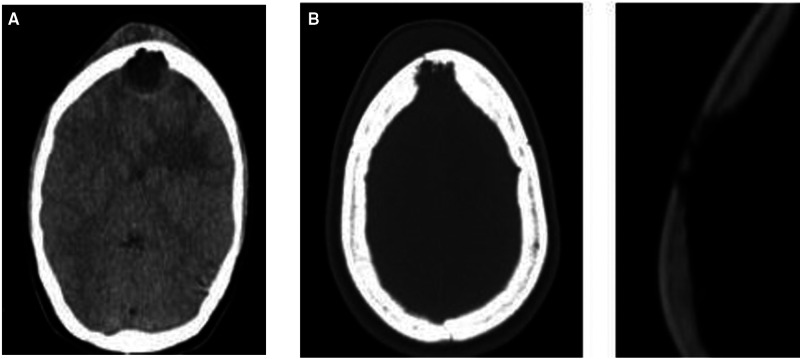
(**A**) Cranial CT, without contrast, axial cut, evidencing subgaleal hypoattenuating formation in the frontal region, noting in the same plane, but in intracranial and extra-axial situation, hypoattenuating lesion with expansive character associated with irregularities in the contours of the inner table of the adjacent frontal bone. (**B**) bone window, in the same plane of subgaleal and extra-axial lesions, evidencing in the frontal bone, especially in the internal table lytic destruction with bone discontinuity.

Blood culture was not performed. CT scan of the skull was performed, which showed an expansive hypodense lesion in the frontal region with intracranial extension associated with perilesional edema (Figure 1), and then transferred to the neurosurgery department with suspicion that the identified expansive formation would have neoplastic origin.

**Figure 2:**
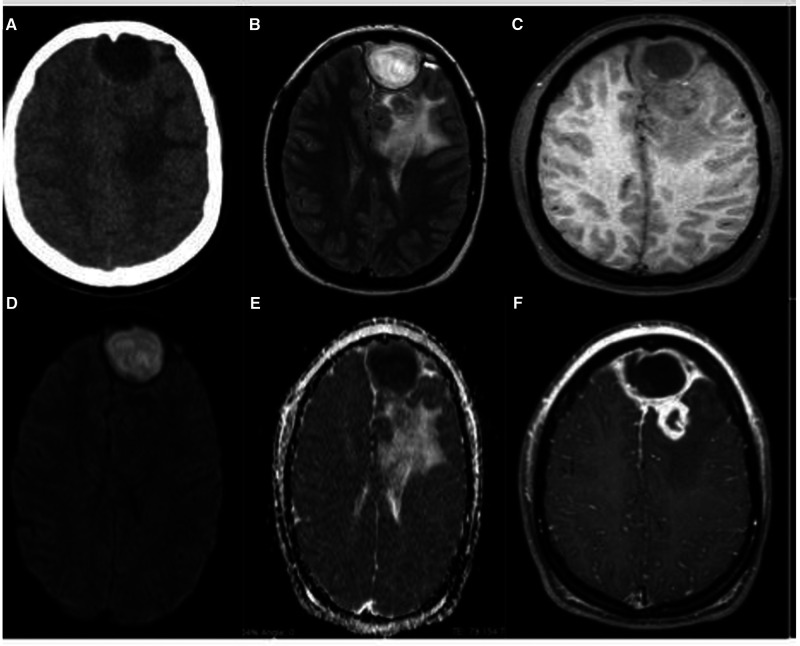
(**A**) Skull CT, without contrast, axial section, evidencing frontal extra-axial lesion, in left paramedian situation and hypoattenuation in the white matter of the left frontal lobe; (**B**) brain MRI, T2/FLAIR, axial, shows better the extra-axial expansive formation, located in the left anterior frontal pole, rounded, with apparent capsule, promoting compressive effect on the adjacent parenchyma, besides evidencing in adjacent frontal lobe small intraparenchymal lesion surrounded by important edema; (**C**) MRI of the brain, T1 after axial contrast, with important peripheral enhancement of both the extra-axial lesion (including with important enhancement of the locoregional meningeal plane) and intra-axial; (**D**) (DWI, axial section) evidences important central restriction of the extra-axial lesion with corresponding signal loss on the ADC Map (**E**) being compatible with empyema/abscess.

The patient underwent brain MRI, which revealed an expansive lesion in the frontal subcutaneous tissue, extending through a bone defect in the frontal sinus that communicated with an extra-axial nodular formation with intra-axial component and circumcision edema with important compressive effect, suggesting a complicated PET with concomitant cerebral abscess (Figure 2). Empirical intravenous antibiotic therapy with ceftriaxone (2 g administrated 12 h/12 h) and metronidazole (7,5 mg/kg administrated each 8 h) was initiated, for eight days before the surgery. Intranasal corticosteroid therapy was not prescribed for our patient.

**Figure 3:**
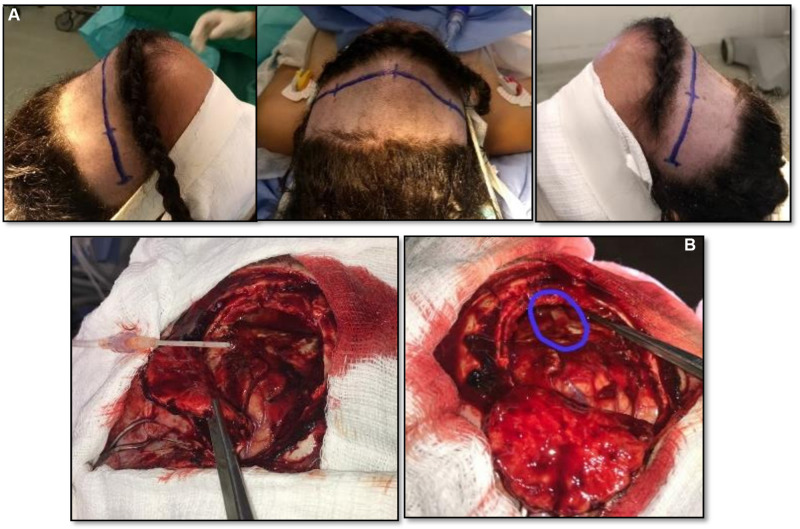
(**A**) Positioning for surgical approach and marking of the bicoronal incision, evidencing the protuberance of the forehead by progressive swelling. (**B**) Intraoperative image after craniotomy with emphasis on fistulous hole of communication with frontal sinus, enhanced.

The patient was submitted to neurosurgical approach by bicoronal incision and, as cutaneous flap was rebalanced, granulation tissue, bone erosion with dural adhesion and spontaneous extravasation of purulent secretion was identified. Drainage of the abscess by puncture initially. Performed 5 × 5 cm frontal craniotomy resecting the entire osteomyelitis area. Resected the residual abscess and capsule, a fistulous hole (Figure 3B) was verified communicating purulent contents of the frontal sinus with brain abscess. After cranialization of the frontal sinus, evacuation of the subdural empyema and subgaleal abscess, exhaustive cavity washing was performed with 1,500 mL of saline plus gentamicin. Cranial reconstruction performed with autologous bone part, after curettage and drill of the internal eroded plate of the frontal bone flap and resected material (pus, granulation tissue and frontal sinus mucosa) sent for microbiological analysis and culture, but it was not identified any organism’s growth. This sterile culture result was probably due to empirical use of preoperative antibiotics, but anaerobic causative agents, destroyed after contact with oxygen, are also a possibility. Similarly, access to histological examination of the frontal sinus mucosa and the granulation tissue collected was not obtained. This type of intervention - surgical approach, associated with antibiotic therapy, was determined based on characteristics such as continuous abscess growth, intracranial mass effect, risk of complications, and neurological deficits.

**Figure 4:**
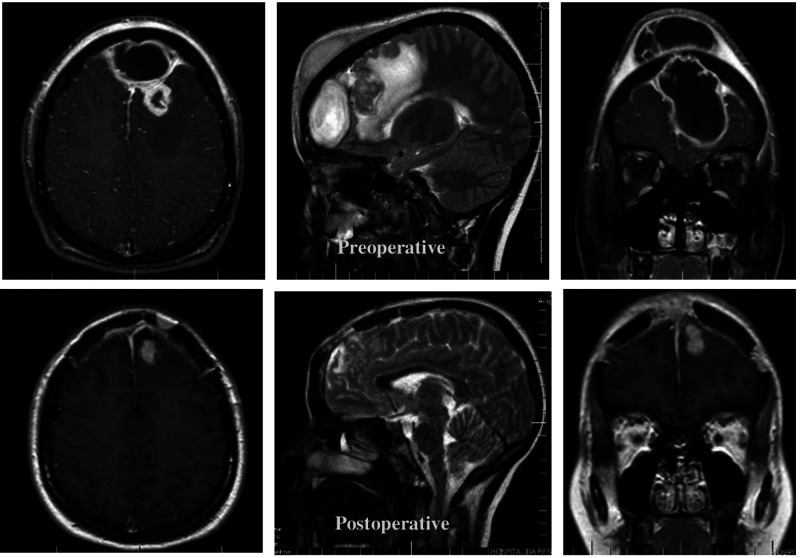
Comparative study between MRI in axial, sagittal and coronal sections before and after the surgical approach.

The patient followed a prolonged course of intravenous antibiotic therapy, ceftriaxone, and metronidazole (7 weeks). A new MRI was performed, 28 days after surgery, and showed the surgical a marked reduction of the frontal extra-axial collection. Meningeal thickening and residual frontal abscess surround by perilesional edema were observed, as shown in the pre and postoperative comparative images in Figure 4.

Due to the significant improvement of our patient condition, she was discharged after 43 days of post-surgical follow-up, with complementary oral antibiotic therapy with ciprofloxacin. It was chosen due to their broad spectrum, low cost, affordability and recommendation by the Hospital Infection Control Committee. The patient was subsequent outpatient follow-up at the service during the first 9 months, in three consultations, with satisfactory clinical, functional, and radiological control. Diagnosis and hospital therapy did not involve an otolaryngologist, but the patient was referred to a professional in the area.

## Discussion

PET is a subperiosteal abscess of the frontal bone as a result of osteomyelitis of the frontal sinus walls (1). Episodes of cranial osteomyelitis are considered rare and are most commonly seen in countries with reduced health capacity, although an increase in the number of cases reported from the second millennium has been observed, most likely due to the improvement in the diagnostic capacity of radiological examinations (5).

Many papers details case reports of PET, and the number of children affected, and the similarity of the associated signs and symptoms are quite relevant, especially in correspondence with the affected locations and the consequent surgical intervention, as described in Table 1. Although this condition can affect both sexes and any age group, reviewing 24 case reports on Pott’s Puffy tumor in patients up to 18 years of age published in the last 5 years, we found reports of patients between 5 and 17 years of age (22 males and 2 females), and observed that there is a higher prevalence among adolescents, in an average age group of 11 years, and in males, as could be noted in Table 1, corresponding to 70% of cases (4). This occurs by the pneumatization process, venous drainage and the peak blood circulation in diploic veins, which occurs around 15 years of age (28). In addition, brain abscess is a complication found in only 12% of patients with PET, and is therefore a relevant fact in the case discussed (4).

**Table 1:** Review of case reports about Pott’s Puffy tumor in patients up to 18 years old.

Author/Year	Age/Sex	Medical history	Signs and symptoms	Features	Pathogenic agent	Drug treatment	Surgical intervention	Outcome
Tibesar, R.J. (6)	15 / Male	Not Mentioned	Nasal obstruction, headache, congestion, and facial pain. Right eye and forehead swelling	Fluid collection over the right frontal bone and cortical breakdown on anterior table	Streptococcus intermedius	Vancomycin, ceftriaxone, and metronidazole	Drainage of the forehead and the right orbital subperiosteal abscess. Frontal sinus trephination	Complete recovery.
Hassan, S. (7)	15 / Male	Facial injury from a basketball	Proptosis and eyelid edema. Fever and inferior hemorrhagic chemosis with mild prolaps	Left extraconal fluid collection, frontal bone osteomyelitis, continued paranasal sinus disease, and a tiny epidural abscess over the frontal lobe	Fusobacterium nucleatum	Vancomycin and ampicillin-sulbactam / ceftriaxone and metronidazole	Orbitotomy and superotemporal abscess drainage and left ethmoidectomy, uncinectomy, and frontal sinusotomy with trephination	The patient had a complete improvement
Joo, M.J. (8)	07 / Female	Nothing noteworthy	Headache and left-sided upper eyelid redness with periorbital swelling	Left-sided frontal sinusitis and osteomyelitis outer table (left frontal sinus) with associated cellulitis of the supraorbital soft tissues	Not Mentioned	IV ampicillin/sulbactam	Procedure not described	Discharged home without further complications.
Miri, A. (9)	14 / Male	recurrent (MSSA) nasal infection, and episodes of facial cellulitis	Frontal headache and forehead swelling. Fever, malaise, and mild cough. Bilateral eyelid edema, and nasal vestibule with dried blood and excoriations	Frontal subgaleal abscess, early osteomyelitis, mild frontal dural meningitis, bilateral preseptal cellulitis, and chronic paranasal sinus disease	MRSA and group A Streptococcus	Cefepime, vancomycin, and metronidazole	Bilateral maxillary antrostomy, right total ethmoidectomy, left anterior ethmoidectomy, bilateral frontal sinusotomy. Drainage of PET	Brain and orbit resolution of dural enhancement and abscess
Stoddard, T. J. (10)	13 / Male	Seasonal allergies	Headache, forehead swelling and fever	Right subperiosteal abscess in frontal sinus and right frontal epidural abscess	Microaerophilic Streptococcus species	Vancomycin, ceftriaxone, and metronidazole	Right craniotomy and epidural abscess evacuation	Patient improvement with any long-term sequalae
Patel, A. (11)	13 / Male	Ear pain and sinuses after scuba diving	Frontal headache and left forehead swelling, periorbital edema, malaise, and emesis	Opacification of frontal sinuses and left anterior ethmoid air cells; frontal subperiosteal abscess and dural enhancement	Negative cultures for growth of organisms	Ceftriaxone, vancomycin, and metronidazole	Incision and drainage of the left frontal subperiosteal abscess, bilateral endoscopic frontal sinusotomy, bilateral endoscopic anterior ethmoidectomy, bilateral maxillary antrostomy	Full recovery without any further complications
Costa, L. (12)	13 / Male	Repeated superior airway infections	Fever, headache and photophobia, right periorbital swelling, cellulitis around the right eye and frontal right tumefaction	Pansinusitis with frontal subcutaneous abscess, inter-hemispheric empyema, and right orbital abscess	No microbiological results	Vancomycin, ceftriaxone, and metronidazole	Drainage of frontal subcutaneous abscess and frontal sinus trepanation, frontal craniotomy with drainage of orbital abscess and interhemispheric and frontal empyema	The patient evolved favorably
AlSarhan, H. (13)	14 / Male	Nothing noteworthy	Headache and upper respiratory tract infection. Fever and right-sided frontal swelling.	Opacity of the left maxillary and frontal sinuses and ethmoid air cells. Osteomyelitis in the frontal and right parietal bones and subgaleal abscess	Staphylococcus aureus and Peptostreptococcus micros	Vancomycin and meropenem	Aspiration of the right frontal swelling. A right eyebrow incision to evacuate the pus. Trephining of the left frontal sinus was performed	The patient improved and no adverse events were reported
Olmaz, B. (14)	12 / Male	Not Mentioned	Generalized headache, enderness at the forehead region and a fluctuant subcutaneous mass	Sinusitis in frontal, ethmoid and right maxillary sinuses with stranding of fat planes in supraorbital subcutaneous tissue	Not Mentioned	Ceftriaxone	Bicoronal skin flap incision with drainage of abscess cavity. A craniectomy to remove infected bony segments was performed.	Not Mentioned
Podolsky-Gondim, G.G. (15)	14 / Male	Obesity, asthma and a chronic use of steroids	Left forehead bulging	Epidural and frontal periosteal abscess. Bony erosion of the internal wall of the frontal sinus	Peptostreptococcus species	Ceftriaxone/ oxacillin/metronidazole and oral amoxicillin with clavulanic acid	Bicoronal skin incision and left frontal craniotomy for drainage of the abscess. The left frontal sinus was cranialized and an extensive removal of debris	Favorable outcome upon long-term follow-up
Öztürk, N. (16)	15 / Male	Not Mentioned	Headache and swelling in both eye and forehead	Erosion in the anterior wall of the frontal. Collection and a sclerotic lesion in both frontal sinuses with development of subperiostal abscess	Not Mentioned	Ceftriaxone and teicoplanin	Procedure not described	Not Mentioned
Linton, S. (17)	16 / Male	Nothing noteworthy	Right frontal swelling and headache. Fever, right orbital proptosis, chemosis, decreased extraocular movement and double vision	Right frontal sinusitis with bony erosion, subperiosteal collection extension into the lateral wall of the orbit	Not Mentioned	Coamoxiclav, metronidazole and otrivine nasal decongestant	Supraorbital incision to exploration and drainage of the right subperiosteal frontal and superotemporal orbital abscess	Improvement in proptosis and diplopia, but visual acuity remained poor
Reddan, T. (18)	06 / Male	Nothing noteworthy	Swelling of the right forehead and headache	Opacification of the right paranasal sinuses, subperiosteal collection	Not Mentioned	Not Mentioned	Procedure not described	The patient evolved favorably
Ikoma, N. (19)	12 / Male	Not Mentioned	Fever and painful forehead swelling	Epidural abscess and air between the frontal bone and superior sagittal sinus and fluid in the right sinuses	Not Mentioned	Meropenem and vancomycin / ceftriaxone and metronidazole	Removal of frontal bone and the drainage of epidural abscess and affected sinuses.	Not Mentioned
Sharma, P. (20)	08 / Female	Sinusitis	Persistent headaches and frontal swelling	Frontal sinusitis, frontal bone defect, and frontal epidural collection - epidural abscess	Streptococcus intermedius	Not Mentioned	Craniotomy with trephination and drainage of the brain abscess. Left ethmoidectomy and frontal and maxillary	Resolution of the subperiostealand epidural abscesses
Nourkami-Tutdibi, N. (21)	06 / Male	Frontal sinusitis	Frontal swelling	Osteomyelitis of the right frontal bone with subdural abscess formation and perifocal edema	Streptococcus intermedius	Cefotaxime and clindamycin	Procedure not described	Good condition without neurologic sequelae
Zamor, R. (22)	15 / Male	Epstein-Barr virus serologies and a monospot positive	Fevers, myalgias, vomiting, diarrhea, cough, headache, and left-sided facial swelling	Left frontal, maxillary, and anterior ethmoid sinusitis. Left orbital cellulitis and subperiosteal collections of the left frontal bone - bone osteomyelitis	Streptococcus anginosus and Fusobacterium necrophorum	Ceftriaxone, clindamycin, vancomycin, and Flagyl	Left lateral orbitotomy, sinus wash-out, drainage of the subperiosteal abscess, and subgaleal drain placement	Patient discharged with follow up of Infectious Disease team
Ling, C. (23)	09 / Male	Not Mentioned	Fever, frontal headache, and frontal swelling	Frontal bone osteomyelitis with subperiosteal collection and frontal sinusitis	Not Mentioned	Not Mentioned	Surgical intervention was not preformed	Complete recovery
Sheth, S.P. (24)	15 / Male	Patient had inserted rugby turf up his nares to stop epistaxis	Photophobia, altered mental status, and right upper extremity weakness. Headache, fever, and emesis. Periorbital erythema and forehead swelling	Frontal sinus subdural empyema, midline shift and mass effect on the cerebral cortex. Presence of left ethmoid foreign body and maxillary sinus opacification	Fusobacterium necrophorum	Vancomycin, ceftriaxone, and piperacillin-tazobactam /metronidazole and meropenem	Left fronto-temporal-parietal craniotomy for subdural empyema drainage. Endoscopic ethmoidectomy with drainage from the left ethmoid, left maxillary sinus, and left nares	Slight difficulty with speech and right-hand coordination difficulty
Sheth, S.P. (24)	17 / Male	Recent sepsis associated with emesis and diarrhea	Fever, left eye swelling, emesis, and diarrhea. Left-sided forehead mass	Left frontal sinusitis, soft tissue edema and early abscess. Subdural empyema in the anteromedial left frontal interhemispheric region	Fusobacterium nucleatum	Vancomycin and metronidazole	Craniotomy for evacuation of subdural empyema and drainage of the left subperiosteal abscess. Left nasal endoscopy with frontal recess exploration drainage	Complete resolution of symptoms on follow-up
Sheth, S.P. (24)	14 / Male	Frontal sinus tenderness.	Eyelid swelling with associated visual changes	Left frontal extra-axial empyema containing air, and involvement of the sagittal, frontal, and paranasal sinuses	Fusobacterium necrophorum	Vancomycin, Ceftriaxone and metronidazole	Bifrontal craniotomy for intracranial abscess. Drainage of right frontal intraparenchymal abscess and sagittal epidural abscess	Complete resolution of symptoms on follow-up
Cannon, L. (25)	05 / Male	Recent sinus infection	Fever, unsteady gait, headache, photophobia, progressive forehead swelling and vomiting	Subgaleal abscess with communication to the frontal sinuses as well as osteomyelitis of the frontal bone	Streptococcus anginosus	Not Mentioned	Drainage of the abscess with evacuation of purulent fluid	Patient improved following surgery
Pérez-Yepes, C.A. (26)	13 / Male	Nothing noteworthy	Headache, fever, and vomiting. Left fronto-orbital oedema	Frontal epidural empyema, left pansinusitis and frontal soft tissue involvement	Group C β-Hemolytic Streptococcal	Cefotaxime, vancomycin and metronidazole	Craniotomy for drainage of epidural and subgaleal collection. Endoscopic surgery of the nose and paranasal sinuses - pansinusitis	Not Mentioned
Nastovska, R. (27)	15 / Male	Frontal sinusitis	Left forehead swelling and tenderness	Scalp abscess, frontal bone osteomyelitis and underlying resolving sinusitis	Streptococcus anginosus	Benzylpenicillin	Abscess drainage and bilateral endoscopic sinus surgery	Not Mentioned

The most commonly cause of PET reported in the literature is acute or chronic sinusitis, which, when treated inappropriately, can culminate in a bone erosion caused by direct contact of the infected material with the walls of the skull cap (28). In addition to this more obvious route of dissemination, there is still the possibility of hematogenous infection through the diploic veins, which, due to the absence of valves, allow a retrograde septic thrombophlebitis more easily, which would explain parenchymal intracranial lesions and subdural collections without epidural involvement (29). Although less frequent, another important cause of PET is craniofacial trauma with a history of fracture. In adults, cocaine or methamphetamine abuse is also reported (2). In our review of case reports, we found that patients had recent or recurrent sinusitis or upper airway infection as the most frequent antecedent (reported in 6 cases), while we found a report of trauma in only one patient.

In addition to the characteristic association with traumatic brain injury and medical history of sinusitis, the presence of more prevalent infectious agents is also well-described for PET. Bacteria’s of rhinogenic pathologies, such as community-acquired sinusitis, are the most common, being *Streptococcus* and S*taphylococcus* the most recurrent agents, such as *Staphylococcus aureus*, *Streptococcus spp*, and anaerobic (30), although fungal infections can also be found. This prevalence can be analyzed on Table 1, which, despite the small amount of data collected, presents 11 (73.3%) cases of *Streptococcus*, among those that reported the causative microbiological agent.

In our review, the most frequently reported symptom is forehead swelling (present in 21 reports), followed by headache (present in 17 reports), fever (present in 13 reports) and symptoms associated with orbital and/or periorbital involvement (present in in 12 reports). As shown in Table 1, it’s notorious that the classic manifestation of this conditions is frontal swelling, usually accompanied by headache and fever. Furthermore, periorbital swelling and pain may suggest cellulitis, being important signs for PET due to possible involvement of the orbital cavity, in addition to purulent or not rhinorrhea (2–4, 28, 29). Symptoms such as nausea, vomiting, changes in the level of consciousness and convulsions may alert to intracranial involvements, such as epidural abscesses, subdural, cerebral, meningitis and thrombosis of the upper sagittal sinus (4). The recommended imaging test to confirm the diagnosis is cranial CT, as it allows a detailed visualization of bone involvement to assess the degree of osteomyelitis. For suspected intracranial lesions, MRI has a greater ability to reveal information, with greater accuracy for soft tissues, it is possible to better quantify the extent of frontal abscesses, for example (2).

In view of the signs and symptoms characteristic of this condition include above all swelling of the frontal region, fever, periorbital swelling, and headache, among the differential diagnoses are those that have mass effect, as well as PET. Thus, the analysis of the presence of soft tissue or skin infection without the presence of osteomyelitis, hematomas and other types of tumor formations should be investigated and ruled out in suspected PET, since they are the main conditions of differential diagnosis (31).

The approach should be based on a combined early intervention of antibiotic therapy and surgical procedure. Initially, the use of broad-spectrum antibiotics is recommended due to the variety of pathogens related with this condition. After the culture result, more specific coverage should be adopted, with the prescribing time ranging from 6 to 8 weeks after surgery (4). The procedure performed can be done from traditional craniotomies, as well as through endoscopic alternatives (32). However, in cases of important lesions, such as brain abscesses, it is mandatory to choose craniectomies with removal of the affected bone, since a high recurrence rate was observed in cases of bone replacement (28, 29). In most of the case reports reviewed, the treatment involved a surgical approach associated with antibiotic therapy, and the majority of patients evolved with a good outcome after treatment, with a description of recovery without sequelae in 14 reports (Table 1).

## Conclusion

Although rare, PET is still present in the pediatric, adolescent and less frequent adult population. The need to rapid recognize and prevent, this sometimes, fatal complication of an apparently benign infection such as bacterial sinusitis, is necessary and mandatory for optimal outcome.

Parents, general clinicians, and pediatricians should be aware of patients presenting recurrent sinusitis, fever, forehead swelling and headache to seek specialist evaluation. Early diagnosis allows for a less invasive approach but in complicated cases, with bone and parenchymal involvement, surgical intervention is crucial to effective treatment, recurrence prevention, and deformity correction. The best management is showed to be a combination of surgical intervention and prolonged intravenous antibiotics.

Suspected by the physician in the context of known risk factors and underlying causes, both clinical and psychosocial, should be searched. In addition, it is a condition that should be readily recognized by the risk of suspected psychosocial clinical complications. An integrated approach is crucial for efficient care.

## Data Availability

The original contributions presented in the study are included in the article/Supplementary Material, further inquiries can be directed to the corresponding author/s.
